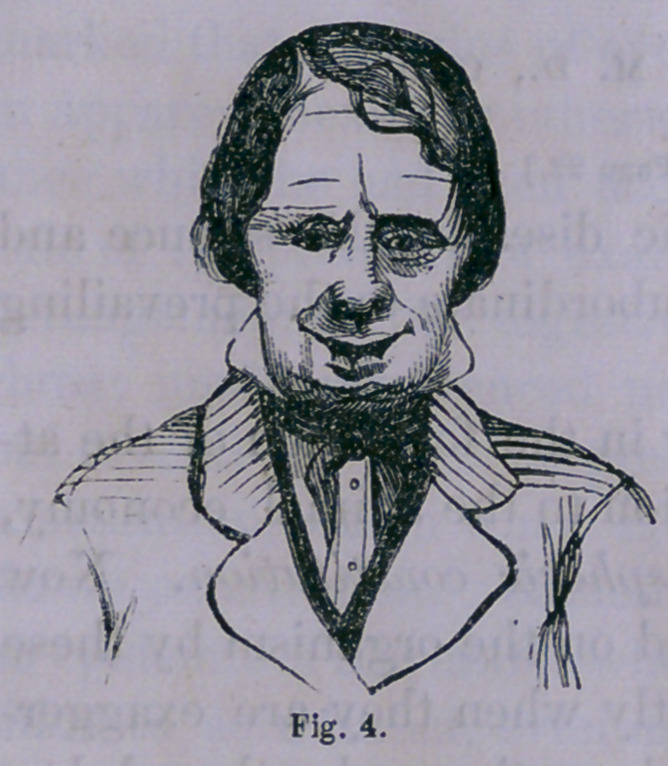# Cases of Disease of the Lower Jaw, for Which Resection and Removal of the Parotid and Submaxillary Gland Was Performed

**Published:** 1860-02

**Authors:** Daniel Brainard

**Affiliations:** Prof. of Surgery in Rush Medical College, Corresponding Member of the “Society of Surgery,” Paris; of the Medical Society of the Canton of Geneva, Switzerland, Etc.


### ARTICLE 2.

### CASES OF DISEASE OF THE LOWER JAW,

### FOR WHICH RESECTION AND REMOVAL OF THE PAROTID AND SUBMAXILLARY GLAND WAS PERFORMED.

BY DANIEL BRAINARD, M. D.,

Prof. of Surgery in Rush Medical College, Corresponding Member of the "Society of Surgery," Paris; of the Medical Society of the Canton of Geneva, Switzerland, etc.

The removal of parts of the lower jaw for necrosis must be an operation as old as the science of surgery. Every surgeon has had occasion to remove pieces of the bone, either its alveolar processes, its body, its condyle, its ramus, or its coronoid processes. I have myself removed all these parts, and some of them many times. Neither is the removal of the whole of the bone when necrosed, a very new, rare or difficult operation. As early as 1817, a case was reported in the Memoirs of the Academy of Surgery. Since that time, numerous instances have occurred when it has been repeated. I have examined the jaw removed by Dr. Carnochan, and found that the body and chin necrosed. In the case of Dr. James Wood, the whole of the bone was, I believe, in that state.

The removal of a considerable part of the bone for disease, whether enchondroma, osteo-sarcoma, or other tumors, is quite another and more difficult operation. I am unable to say by whom it was first performed, but the credit is generally given to Dr. Diedrich, of Kentucky. Dupuytren performed it, it is said, as early as 1812. And Dr. Mott, in 1822, removing a much larger portion. Since that time, cases are counted by hundreds, and there are few surgeons who have not contributed more or less of them.

I have three times dis-articulated the lower jaw, removing twice a little less than half, and the other just half of it. In each case, the parotid gland, or what remained of it after being subjected to the pressure of the tumor, was removed.

Case 1. This was published in the American Journal of Medical Sciences for October, 1853, but is re-published here on account of the local interest which is at this moment attached to the subject among a small circle of physicians in this city.

"W. S., æt. 32 years, formerly of good constitution, first perceived in October, 1850, that the movements of the lower jaw were imperfect. Soon after, he noticed a swelling at the left side of the articulation. It was hard, unattended with pain or discoloration, and increased slowly. In February, 1852, an abscess formed upon the side of the neck, below the angle of the jaw, from which, when opened, a fistulous passage leading to the bone, remained. Some months afterwards, another abscess formed in front of the ear, from which pus and saliva were discharged. Another formed upon the zygoma. During the whole of this time, the patient's health was not materially impaired.

He was treated principally with different preparations of iodine, internally and externally.

Present state, Jan. 19, 1853. There is a hard tumor, extending from the temple above, below the angle of the jaw, and from the middle of the cheek to the ear. It is very prominent and irregular, and has on its surface three fistulous openings, corresponding to the situation of the abscesses. They were found to converge toward the articulation. The mouth could be but slightly opened, but enough to enable me to perceive that the tumor encroached upon the fauces and pharynx. There is pain most severe at night. When he eats, the saliva flows freely from the middle fistula. His health is good, no sallowness, and only a little emaciation, which dates from about a month.

Operation performed Jan. 27, 1853, with the assistance of Drs. Herrick, Freer, and Morfit, as follows:

The patient having been placed under the effect of chloroform, a curved incision was made, commencing an inch above the zugoma, carried down in front of the ear over the angle of the jaw, and brought forward as far as the side of the chin. The flap was raised, and the integuments dissected up from the surface of the tumor behind.

The bicuspid tooth of the same side was extracted, the tissues detached from the jaw, and the bone divided with a chain saw.

At this stage of the operation, it was necessary to allow the effects of the chloroform to pass off, as the flow of blood into the mouth and throat rendered it necessary that it should be constantly ejected.

The separation of the tumor below and within was next effected, as far back as the mastoid process, against which it was placed.

An attempt was now made to turn it upwards, but it was still almost immovable, and it was necessary to attack it from above.

The temporal muscle was divided, the zygomatic arch cut away at its anterior and posterior extemities, the masseter muscle divided, and a chisel and mallet used to separate the articulation; but the bones were so confounded together, that a part of the glenoid cavity was cut away. A lever being inserted behind the tumor, strong efforts were made to turn it forward, but without success. It was then attacked from within; the pterygoid muscles, which were semi-cartilaginous, were divided at their attachment to the pterygoid processes. The mass was then raised from its position, and its remaining adhesion separated.

The vessels which gave blood were very numerous. The facial, lingual, external carotid, and numerous smaller branches, required the ligature.

The ninth pair of nerves, the portio dura, and the dental branch of the lingual were divided.

On examining the wound, it was found to lay bare the cornu of the os hyoides, the mastoid and styloid processes, and the transverse processes of the cervical vertebræ. The internal jugular vein and the internal carotid artery were seen at the bottom of the wound. The temporal fossa was excavated, the muscles of the tongue and pharynx laid bare. The parotid gland had been partially obliterated by pressure, and no trace of it now remained.

The wound was brought together by stitches, and cold water dressings applied. A full dose of opium was administered. The operation, which lasted an hour, was well borne.

*After treatment.* No symptoms requiring especial notice occurred. The ligatures were all removed by the tenth day.

The wound had adhered throughout, excepting at the point where the ligatures prevented.

Dec. 5. The patient's health is good. He can sit up and walked about, but requires an anodyne at night, on account of pain.

July 17. There still remains a fistulous opening, leading to the situation of the glenoid cavity, through which a small piece of bone has been discharged. The lower jaw has fallen so much to one side that the patient cannot masticate. He is emaciated, and appears to be suffering from inanition, resulting from insufficient nourishment. He still requires anodynes to procure rest at night. There is no appearance of return of the disease.

On examination of the tumor, the bone seemed greatly enlarged in every sense, the ramus of the jaw and the angle were four times their natural thickness, the condyle partially absorbed and altered in shape. This mass, which had the appearance of bone, and from which the periosteum could readily be separated, was so soft as to be easily transfixed with a sharp instrument. The surrounding tissues were infiltrated with serum, and had an appearance like firm jelly, but after maceration, presented no appearance of morbid deposit."

Case 2. Silas Lattimer, American, aged 27 years, applied at the college clinic, Nov., 1857, on account of an enlargement of the left side of the lower jaw. It had been first perceived about two and a half years previously, when the teeth of that side became painful, loosened, and were removed. By degrees the tumor increased in size until it assumed the appearance presented in fig. 1.

The operation was performed on the 28th November, in the same manner as the preceding case, except that a little more than half the jaw was removed, including the condyle and symphysis. The patient bore the operation well, although from the liability of the blood to flow into the air passage, chloroform could be but imperfectly used. There was considerable tendency to suffocation from the falling back of the tongue on the division of its anterior attachments, but this soon ceased. He did well, and was able to return home Dec., 7th, with the wound nearly healed.

On examining the tumor, it was found to contain a soft, brain-like matter, which seemed to have been developed in the centre of the bone. It exhibited the cancer cells in a very marked degree of development with the microscope. The cavity left corresponded, in every respect, with that in the preceeding case, and the same vessels and nerves were divided.

Fig 1.

March 12, 1858. I received the following note from him, accompanied by a daguerreotype, from which Fig. 2 is engraved. It will be seen that there remains but little deformity, although the muscular nerve of the face was necessarily denuded.

Fig. 2.

WILMINGTON, MARCH 12, 1858.

DR. D. BRAINARD: Sir—I send you my figure, which was taken March 8th. The cut is not entirely healed yet. Two pieces of bone came off the end that was sawed off. I think the *disease* was all taken out, as the slivers appeared sound.

Yours with respect, SILAS LATTIMER.

CASE 3.—Mrs. C., aged 31 years, consulted me April 28th, 1858, on account of a tumor situated on the lower jaw. The appearance of it at this time is well represented in the figure. It follows the natural outline of the jaw extending from the zygomatic arch to the middle of the base of the jaw, backward to the mastoid process. It was of stony hardness, not painful nor discolored.

Fig. 3.

*History.*—In November, 1857, Mrs. C. had a *tooth-ache*, for which the wisdom tooth was extracted, after which considerable swelling of the whole of the right side of the face occurred, accompanied with much febrile reaction. When this subsided some enlargement of the bone remained, this has gradually increased until the present time.

*Operation.*--This was performed May 1st, 1858, with the assistance of Doctors Powell, Durham, Winer and several students. The method followed was so nearly that adopted in the preceding cases that details are unecessary. The zygoma was removed, the pteregoid muscles were divided close to their attachment to the processes. The tumor was so closely adherent to the styloid process, that this broken, the parts were very vascular and many ligatures were required. The external carotid artery was divided and tied.

August 30. Wound nearly all healed by the first intention, patient taking food freely and free from pain.

September 8. Returned home in good health and with little deformity. Figure 3 represents her appearance at that time. At the present time of writing, this March, 1858, there has been no return of the disease.

I have removed parts of the lower jaw for tumors of various kinds, in a large number of cases, of several of which no record is preserved. The following two cases are among the most remarkable.

Case 17.—Ole S——, Norwegian, aged about 45, entered the Mercy Hospital August 23d, 1855, with a cancerous tumor involving the anterior part of the lower jaw. The history of the case as far as could be learned, was as follows:—About one year previously he had a pain and looseness in the incisor teeth of the lower jaw, for which he had several extracted, soon after there sprung up from the several sockets, a fungus growth which was cut away, this was speedily reproduced, and about six months prior to his presenting himself at the Hospital this was removed by an operation, the alveolar process, and a part of the upper edge of the jaw being removed by a saw. The wound cicatrized and for a time a cure seemed to have been effected, but in a few weeks the disease reappeared, and at the time of his entrance into the Hospital presented the appearance of an enlargement of the bone in every direction, pushing back upon the tongue and rendering swallowing difficult. Upon the upper surface were several holes and irregularities exhibiting unmistakable signs of cancerous humor to the present time.

Fig. 4.

*Operation.*—An incision was made from one angle of the jaw to the other, along its base and across the chin, one flap was dissected upwards and another downwards, the mouth being laid open by the former dissection. Section of the bone was made on each side by means of the chain saw, behind the last molar tooth. The hemorrhage was severe and eight arteries required ligature. At the moment of severing the anterior attachments there was a tendency to strangulation from the tongue falling backward, this was relieved by an assistant holding the tongue forward for a time, and afterwards by placing the patient on his face, which position he was obliged to keep for several days, particularly when sleeping.

The patient was kept fully under the effect of chloroform, and bore the operation well.

On cutting into the tumor it was found to consist of a firm mass surrounding the bone resembling schirru, cracking under the knife. No cancer cells were found by the microscope, fibres, blood globules and granular matter, which seemed like coagulated albumen, were the principal elements observed.—At the point where the tooth had been extracted, there was a considerable cavity containing reddish pus and blood, otherwise the tissue of the bone was but little altered.

In this case not only the part of the jaw as far forward as the biscuspid tooth, but all the parotid and sub maxillary glands were removed. They were both diseased, and so firmly adherent to the tumor that they could with difficulty be separated from it. The parotid particularly found a part of the diseased mass.

## Figures and Tables

**Fig 1. f1:**
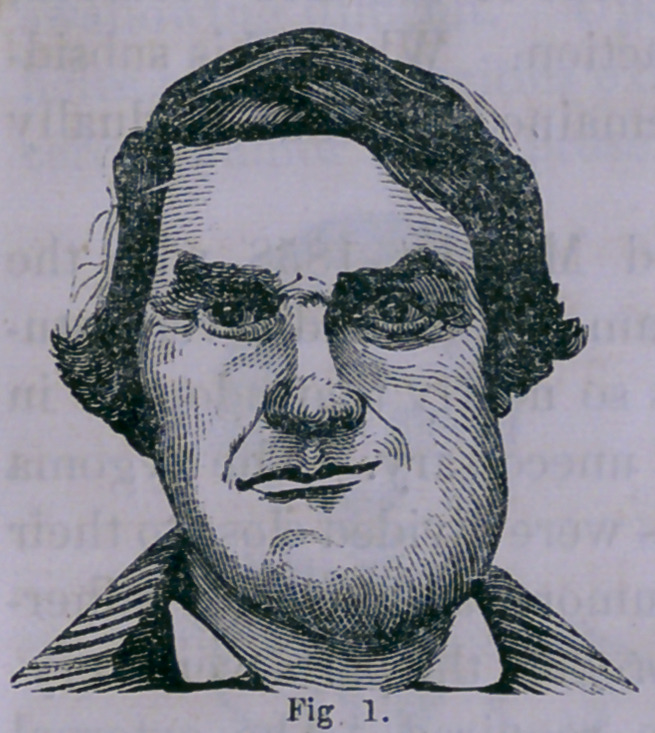


**Fig. 2. f2:**
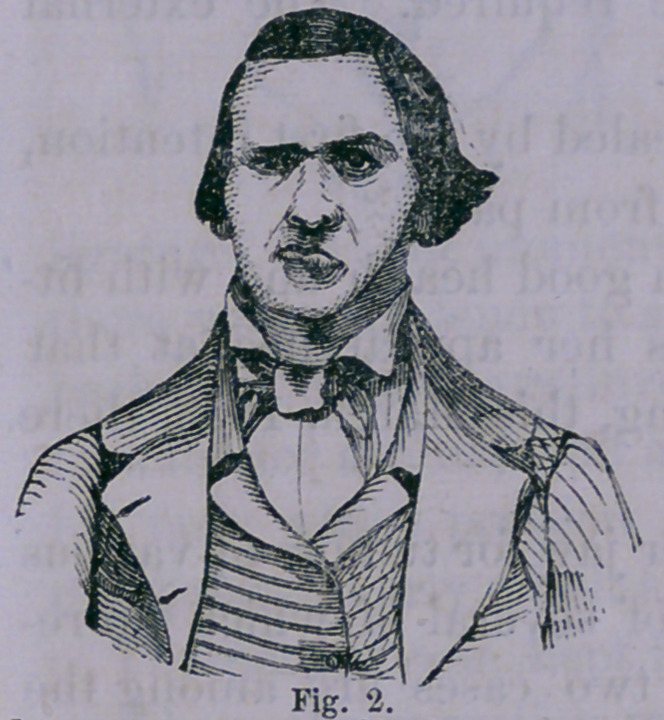


**Fig. 3. f3:**
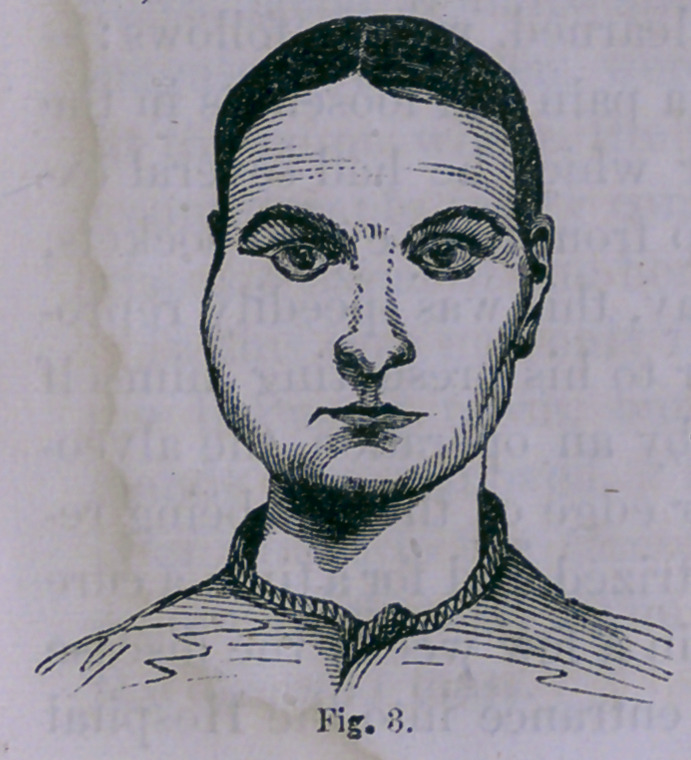


**Fig. 4. f4:**